# AI-predicted zona pellucida binding: shortcut learning and clustered validation may overstate clinical portability

**DOI:** 10.1093/hropen/hoag069

**Published:** 2026-08-03

**Authors:** Tong Wang, Yue Li, Ran Tong

**Affiliations:** Department of Biology, Duke University, Durham, NC, USA; College of Physics and Optoelectronic Engineering, Shenzhen University, Shenzhen, China; Department of Computer Science, Purdue University, West Lafayette, IN, USA; Department of Mathematical Sciences, University of Texas at Dallas, Richardson, TX, USA

Dear Editor,

We read with interest the *Human Reproduction Open* article by Leung *et al.* (2025), which developed a deep-learning model to identify human spermatozoa with zona pellucida (ZP)-binding capability. The study is innovative because it moves AI-based sperm assessment beyond World Health Organization (WHO) morphology grading (Pham *et al.*, 2025) and towards a functionally relevant fertilization phenotype. However, three methodological issues may overstate the model’s clinical utility and should be addressed before the proposed threshold is used to guide IVF versus ICSI decision-making.

First, the training labels may confound ZP-binding biology with image-domain shortcuts. ZP-bound spermatozoa were collected after sperm–ZP co-incubation, whereas ZP-unbound spermatozoa were obtained from normozoospermic samples with complete fertilization failure and absence of ZP-bound spermatozoa. This contrast is biologically meaningful, but it is also procedurally asymmetric. The two classes may differ not only in sperm-intrinsic morphology, but also in sample source, slide preparation, staining context, background texture, debris, and acquisition conditions. In medical imaging AI, such non-biological signals can become shortcuts and contribute to reduced external generalization (Zech *et al.*, 2018). Therefore, high discrimination may partly reflect laboratory-domain separation rather than a pure morphology-based signature of ZP-binding capability.

Second, the validation strategy may not fully address clustering. The study reports clinical validation on >33 000 sperm images, but the independent clinical unit is the man, couple, cycle, slide, staining batch, or laboratory workflow—not the individual sperm image. Similarly, the reported K-fold cross-validation used randomly split image subsets. If images from the same donor, slide, or batch can appear across training and validation folds, model performance may reflect within-sample similarity rather than true generalization to unseen patients. Image-level abundance should not be treated as patient-level sample size. This distinction is critical when a model is intended to influence insemination strategy.

Finally, the CycleGAN step and the fixed 4.9% threshold expose a domain-shift problem. Leung *et al.* (2025) stated that subtle clinical microenvironmental differences could confuse the classifier and used CycleGAN to reduce background noise and improve image consistency. This is technically thoughtful, but it also suggests that model behaviour is sensitive to image domain. If performance depends on image conversion, then staining lot, microscope, camera, slide handling, segmentation pipeline, and laboratory workflow may alter the decision boundary. A threshold derived by Youden’s index in a single-centre workflow should therefore be considered internally optimized, not clinically applicable.

These issues have direct clinical implications. Leung *et al.* (2025) propose that the model could identify couples at high risk of unexpected IVF fertilization failure and allow alternative insemination methods. Such use would affect IVF versus ICSI counselling, patient anxiety, laboratory workload, and possible overuse of ICSI. Therefore, the model should meet a higher standard than internal image classification accuracy.

We suggest four clarifications or additional analyses: (i) report whether data splitting was performed at donor, slide, staining-batch, and clinical-cycle levels, (ii) perform leave-one-donor-out, leave-one-slide-out, and leave-one-batch-out validation, (iii) test the model in external ART laboratories using different microscopes, staining lots, cameras, and image-processing pipelines, and (iv) recalibrate the 4.9% threshold and evaluate decision-curve utility prospectively before using it for insemination decisions.

Such reporting would be consistent with current guidance for transparent AI prediction and medical imaging AI studies (Mongan *et al.*, 2020; Collins *et al.*, 2024).

Leung *et al.* (2025) have provided an important proof of concept for functional sperm image analysis. The unresolved question is whether AI-predicted ZP-binding is a generalizable sperm functional phenotype or a high-performing classifier within a controlled image domain. For ART, the key issue is not only whether an algorithm can recognize ZP-binding-associated images, but whether its prediction remains reliable when the patient, slide, staining protocol, microscope, and clinical decision context change.
